# Validity and Reliability of the Berg Balance Scale in Different Tele‐Assessment Methods in Patients With Stroke

**DOI:** 10.1111/jep.70141

**Published:** 2025-06-10

**Authors:** Birol Önal, Nezire Köse, Şeyma Nur Önal, Hatice Yağmur Zengin

**Affiliations:** ^1^ Department of Physiotherapy and Rehabilitation Atatürk University Erzurum Türkiye; ^2^ Faculty of Physical Therapy and Rehabilitation Hacettepe University Ankara Türkiye; ^3^ Bartın University, Vocational School of Health Services, Physiotherapy Program Bartın Türkiye; ^4^ Department of Biostatistics, Faculty of Medicine Hacettepe University Ankara Türkiye

**Keywords:** balance, physiotherapy, stroke, teleassessment, telehealth

## Abstract

**Aims and Objective:**

Balance evaluation is essential for determining treatment and its effectiveness in stroke patients. Considering the widespread use of telehealth services, it is important to evaluate the applicability of balance scales for teleassessment. The aim in this study was to investigate the reliability and validity of the Berg Balance Scale (BBS) applied using synchronous and asynchronous teleassessment methods.

**Method:**

Teleassessments were performed by two physiotherapists. Synchronous assessments were conducted online in real time using the application Zoom, while asynchronous assessments involved patients recording videos according to a reference evaluation video sent to them. All tests were repeated 10 days later to assess intrarater reliability.

**Results:**

Thirty‐six stroke patients (mean age 55.9 ± 9.5 years) participated in the study. Both synchronous and asynchronous teleassessments of the Berg Balance Scale (BBS) demonstrated excellent interrater reliability, with ICC values of 0.989 for synchronous and 0.997 for asynchronous assessments. Intrarater reliability was also high, with ICCs ranging from 0.982 to 0.997 across raters and methods. Regarding concurrent validity, synchronous teleassessment BBS scores showed a strong correlation with face‐to‐face BBS (*r* = 0.970) and Timed Balance Test (TBT) scores (*r* = 0.901), while asynchronous assessments also demonstrated strong correlations (BBS: *r* = 0.945; TBT: *r* = 0.885). Correlations with postural sway parameters were moderate, ranging from *r* =−0.40 to −0.54.

**Conclusion:**

Our findings suggest that synchronous and asynchronous teleassessment of the BBS may be a viable alternative to face‐to‐face assessments. However, further research with larger samples is needed to support these findings and increase their generalizability.

**Trial Registration:**

ClinicalTrials.gov identifier: NCT05263063.

## 1 Introduction

Stroke is a common neurological problem that occurs frequently and leads to disabilities. Globally, it is the third most common cause of disability and the second most common cause of death [1]. As the population ages, the occurrence of strokes is on the rise each year [2]. However, due to the development of treatment techniques and medical interventions, post‐stroke mortality rates are gradually decreasing. This trend is further increasing the need for stroke rehabilitation [3].

In stroke patients, various problems such as hemiparesis, muscle strength loss, spasticity, abnormal muscle activation, and sensory, perceptual, and cognitive issues are observed [4]. Due to all these motor, sensory, emotional, and cognitive problems, patients’ lives are significantly restricted. However, patients commonly report balance and fall problems as the most limiting issues affecting their activity levels [5]. Therefore, to enhance balance and prevent falls, stroke patients should be assessed via reliable and valid methods, and personalized, effective treatment programs should be implemented to address their specific needs.

In the traditional rehabilitation approach, during the rehabilitation process, balance assessments are conducted face‐to‐face in a clinical environment, and treatment plans are drawn up. However, access to physiotherapists and physiotherapy services for stroke patients may be constrained due to issues such as transportation difficulties, a lack of physiotherapy centers in rural areas, environmental factors, financial constraints, or other health problems the patient may have. In such cases, the feasibility of implementing assessment programs for patients becomes challenging, indirectly impacting the ability to establish and implement treatment programs [6, 7]. Teleassessment can provide equal opportunities for individuals with disabilities in such situations [8]. There are two subtypes of teleassessment: synchronous and asynchronous [9]. In synchronous teleassessment, assessments are conducted in real time between patient and therapist using digital tools such as video conferencing [10]. Asynchronous teleassessment refers to a time‐ and space‐independent approach in which the patient's video recordings or previously collected data are later reviewed by the therapist [11]. Synchronous telediscussion provides real‐time interaction and instant feedback, but can entail challenges such as timing and Internet issues [10]. Asynchronous teledelivery offers flexibility and can be performed without time constraints [11]. However, there may be delays in receiving feedback and patients may submit inaccurate or incomplete video recordings [12, 13]. Asynchronous teleassessment may be a more suitable alternative when there are barriers such as busy working hours or time differences [13, 14]. Therefore, it is important to investigate the validity and reliability of both methods, considering their different advantages and disadvantages.

Due to the COVID‐19 pandemic, telerehabilitation, a subset of the telehealth system in which patients are remotely monitored and treated, has gained importance [15]. The literature on telerehabilitation in stroke patients is increasing [16, 17]. In such studies, patients are typically evaluated face‐to‐face, and rehabilitation programs are conducted remotely using telerehabilitation methods [18, 19]. However, studies investigating the reliability and feasibility of teleassessment methods in stroke patients remain limited, despite the clear need for such research [20, 21, 22]. Moreover, the urgent need to develop assessment tools for remote use in rehabilitation for stroke patients in rural areas with limited access to physiotherapy services is emphasized in the literature [20]. In particular, asynchronous teleassessment—which allows assessments to be conducted without real‐time interaction—may offer additional advantages for stroke patients living in rural or underserved areas where scheduling synchronous sessions or ensuring stable internet connectivity can be challenging. Despite these potential benefits, research on the validity and reliability of asynchronous teleassessment methods in stroke populations remains scarce, underscoring a critical gap in the current literature.

The Berg Balance Scale (BBS) is a widely used tool to assess balance, especially in patients with neurological diseases such as stroke [23, 24]. The BBS contains 14 items measuring balance related tasks and is commonly used to estimate the risk of falls in stroke patients [25]. It is important to determine the suitability of the BBS, which is frequently used in clinical practice, for teleassessment. There are studies reported examining the suitability of the BBS for telerating. In one of these studies it was found that the BBS can be reliably rated remotely with video, but anterior and lateral views, high‐resolution video, and slow‐motion examination facilities are required [26]. In other studies, the validity of the BBS was investigated only in stroke patients and their evaluations were based on real‐time video [27]. In our study, the aim was to investigate the validity and reliability of the BBS evaluated by both synchronous and asynchronous teleassessment for chronic stroke patients.

## 2 Materials and Methods

### 2.1 Participants

This cross‐sectional observational study was conducted at the Faculty of Physical Therapy and Rehabilitation, Hacettepe University, between January 2022 and March 2023. Ethical approval for the study was obtained from the Non‐Interventional Clinical Research Ethics Board of Hacettepe University, Türkiye, on January 18, 2022 (Approval No: 21/2117). In the sample size calculation, it was assumed that 2 repeated measurements were obtained from 30 participants to provide 80% power at 95% confidence level for the intraclass correlation coefficient (ICC), with an expected ICC of 0.80 and a minimum acceptable ICC of 0.60 under the null hypothesis. With the assumption of 20% missing patients from the study, 36 patients were included. Our study was reported in accordance with the GRRAS and COSMIN checklists and its methodological quality was assessed [28, 29].

**Figure 1:**
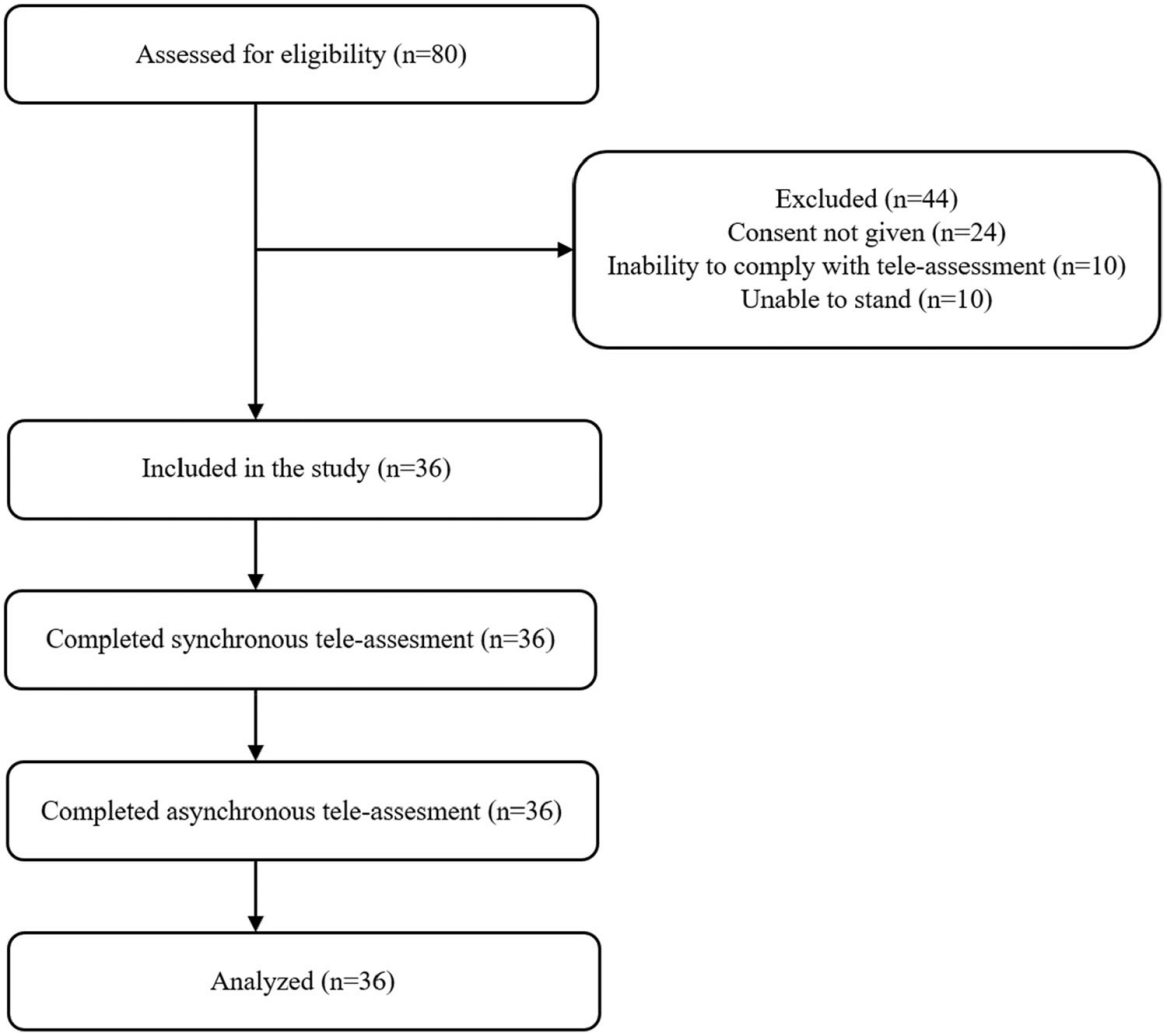
STROBE Flow Chart.

During the data collection process, 80 patients were interviewed. Thirty‐six patients who met the inclusion and exclusion criteria and agreed to participate were included. Patients who were over 18 years of age, had no cognitive or communication problems, had a Brunnstrom lower extremity level of at least 3, could stand, and had experience using technological devices were included in the study. Patients with auditory and visual dysfunction, with neurological diseases other than stroke (multiple sclerosis, Parkinson's disease, ataxia, dystonia, dyskinesia, etc.), or with psychiatric disorders were not included. All participants provided written informed consent before enrollment. They were specifically informed about the procedures and potential risks associated with tele‐assessment, including possible technical difficulties, data privacy considerations, and the absence of immediate physical assistance during remote assessments. A flowchart detailing patient selection in the study is given in Figure 1.

### 2.2 Procedure

Chronic stroke patients who presented to Hacettepe University's Faculty of Physical Therapy and Rehabilitation and met the inclusion criteria were enrolled. Evaluations were performed on three consecutive days. Teleassessments were conducted by two physiotherapists, each with extensive experience in administering the BBS in clinical practice (> 7 years). Before the study, the raters underwent a structured training session to ensure standardization of test instructions, assessment procedures, and scoring criteria. This training included a detailed review of the BBS protocol and discussions to resolve potential ambiguities. The face‐to‐face interview and face‐to‐face evaluation were performed by the first physiotherapist in the clinical environment. In the face‐to‐face evaluation on the first day, the patient's demographic information, cognitive status, stroke severity, Tinetti balance test (TBT), BBS, and postural sway evaluations were performed. The next day, only the BBS was applied using synchronous or asynchronous teleassessment. Evaluations were conducted 1 day apart and at the same time of day. To avoid the effect of learning during the evaluations, the order of the evaluations applied to each new individual was varied. The synchronous teleassessment was evaluated online in real time by the first and second physiotherapists. The patient's phone was fixed on a tripod and the assessment was conducted via the application Zoom. Both physiotherapists performed the assessment during the same session but joined the Zoom meeting on different computers in different locations. During the synchronous evaluation, a video recording of the evaluation was also taken. These video recordings were used for retesting. For the asynchronous teleassessment, a reference evaluation video was sent to the patients. The duration of the reference video was about 4 min. The reference evaluation video was filmed with a healthy 26‐year‐old participant. It was designed to explain how to position the phone, the items needed for the assessment, and the safety precautions to follow before the evaluation. The video provided detailed instructions on how to position the camera and what materials were necessary for the assessment. In addition, safety instructions were included to ensure proper execution of the test. This video was sent to the patients’ relatives via WhatsApp. The relatives recorded their own assessment videos using the instructions provided and sent them back to the research team via WhatsApp. The video was always recorded on a smartphone; no personal computers were used during the recording. The patient's relatives were asked to evaluate the patient by watching this video. They were also asked to record video while they performed these asynchronous evaluations. These video recordings were then evaluated separately by the first and second physiotherapists. Each assessment took approximately 30 min. For reliability, the synchronous teleassessment was performed in 50% of the participants, and the video recordings taken during this initial evaluation were re‐evaluated 10 days later as part of the retest process. Similarly, asynchronous teleassessment videos, recorded during the initial asynchronous evaluation, were re‐evaluated 10 days later in 50% of the participants to serve as the retest for this assessment. No adverse events occurred during either synchronous or asynchronous assessments, confirming the adequacy of the safety measures implemented throughout the study.

### 2.3 Outcome Measures

Demographic and clinical information such as age, height, body weight, occupation, educational status, affected side, type, and duration of stroke were recorded. In addition, the cognitive status of the stroke patients was evaluated with the Mini Mental State Examination [30]; their balance with the BBS, the TBT, and a FreeMed baropodometric platform (Sensor Medica, Guidonia Montecelio, Rome, Italy); and their motor function with the Brunnstrom Stage [31].

Postural sway was evaluated with the FreeMed baropodometric platform. The platform is 60 cm by 50 cm in size and features freeStep software with a sampling frequency of 400 Hz. The participants were asked to place their bare feet on the platform in a “relaxed and natural” position, with about 5 cm between the heels. Using the static balance evaluation module, sways in the x and y axes (DeltaX and DeltaY), and sway speed (Avspeed) were evaluated for 20 s. The x‐axis corresponds to mediolateral sway (MLS) and the y‐axis to anteroposterior sway (APS) [32].

The TBT and BBS were used to evaluate the patients’ balance. The TBT consists of nine items. The items include an evaluation of sitting balance, standing balance, balance evaluation with eyes open and closed, 360‐degree rotation around oneself, and lying down. While seven items are scored from 0 to 2 points, two items are scored as 0 or 1 point. The TBT is scored out of 16 points [33]. The BBS consists of 14 items evaluating tasks frequently performed during daily living activities. It includes functions such as standing from sitting, sitting without support, standing without support, sitting from standing, transfers, standing with eyes closed, standing with legs together, reaching forward while standing, picking up objects from the floor, looking behind over both shoulders, turning 360 degrees, placing alternate feet on a step, standing with one foot forward, and standing on one leg. Each item is scored between 0 and 4 points. The total score of the test is between 0 and 56 [34]. The validity and reliability of the Turkish versions of both tests used in balance assessment have been confirmed [33, 35].

### 2.4 Statistical Analysis

The statistical analyzes were performed using SPSS Version 26.0 (IBM Corp., Armonk, NY, USA). The normality of the numerical data was assessed using the Shapiro–Wilk test. The numerical data for the demographic and clinical features were presented as the mean and standard deviation for normally distributed data. Otherwise, medians (IQR) were presented as descriptive statistics. The categorical data are given as frequency (n) and percentage. The ICC was used to assess intrarater and interrater reliability, and ICC (3,1) values were presented with 95% confidence intervals according to the definition of absolute agreement, considering the two‐way mixed effect model. ICCs were classified according to the study by Alpar et al. (0.95–1.00 = excellent; 0.85–0.94 = high; 0.70–0.84 = fair; 0–0.69 = unacceptable) [36]. To calculate the standard error of measurement (SEM), the difference between the scores obtained during the first and second trials was computed as a variable. The standard deviation (SD) of these differences was calculated and used to determine the SEM using the formula: SEM = $\frac{SD}{\sqrt{2}}$. The SEM with 95% confidence interval (SEM95%) was then calculated as SEM × 1.96. The smallest detectable change (SDC95%) with 95% confidence interval (CI) was determined using the formula SDC95% = SEM × 1.96 × $\sqrt{2}$ [37]. Validity was determined by correlating the BBS scores from the synchronous and asynchronous teleassessments with scores from other balance tests, establishing concurrent validity. Correlations between BBS scores and other balance tests were examined using Pearson's correlation coefficients if the requirements were met. Otherwise, Spearman's rho correlation coefficient was used. The correlation coefficients were interpreted as negligible (0–0.199), weak (0.20–0.39), moderate (0.40–0.59), strong (0.60–0.79), and very strong (0.80–1.00) [38]. The statistical significance level was set at *p* < 0.05.

## 3 Results

The clinical and demographic data for the test and retest groups are presented in Table 1. While the average age of the test group was 55.9 years, the average age of the retest group was 54.9 years. The sex ratio in both groups was 2/1 (male/female). While 25 of the patients in the test group had had an ischemic stroke and 11 had had a hemorrhagic stroke, 10 of the patients in the retest group had had an ischemic stroke and 8 had had a hemorrhagic stroke.

**Table 1:** Demographic characteristics of the particapants.

	Test Group (*n* = 36)		Retest Group (*n* = 18)		
Variables	X ± SD	Median (IQR)	X ± SD	Median (IQR)	*p* ^a^ ^,^ ^b^
Age (yeras)	55.9 ± 9.5	55.5 (50.0–61.0)	54.9 ± 8.6	52.0 (48.0–58.0)	0.382^a^
Height (cm)	168.0 ± 9.1	169.0 (161.5–174.0)	165.9 ± 8.4	165.0 (158.0–174.0)	0.519^a^
Weight (kg)	75.3 ± 12.7	73.5 (65.0–84.5	72.1 ± 11.2	69.5 (65.0–85.0)	0.417^b^
Body mass index (kg/cm^2^)	26.6 ± 3.2	26.4 (24.0– 28.3)	26.0 ± 2.5	25.7 (23.8–27.7)	0.614^a^
MMSE (0–30)	28.2 ± 0.9	28.0 (28–29)	28.0 ± 0.9	28.0 (27.0–29.0)	0.338^b^
MLS (mm)	7.7 ± 6.4	5.8 (3.8–9.9)	9.3 ± 8.1	7.9 (4.0–11.7)	0.199^b^
APS (mm)	5.6 ± 3.3	4.4 (3.1–7.3)	6.2 ± 3.9	4.7 (3.3–7.3)	0.545^b^
Sway velocity (mm/s)	7.4 ± 2.6	6.9 (5.6–9.3)	8.2 ± 6.9	2.5 (5.1–7.1)	0.811^b^
Time since acute event (months)	40.0 ± 35.4	30 (15.0–60.0)	40.8 ± 30.7	30 (18.0–60.0)	0.712^b^

	*n* (%)		*n* (%)	*p* ^c^
Sex	Female Male	12 (33.3)	6 (33.3)	0.616^c^
		24 (66.7)	12 (66.7)	
Type of stroke	Hemorrhagic Ischemic	11 (30.6)	8 (44.4)	0.236^c^
		25 (69.4)	10 (55.6)	
Dominant side	Right	31 (86.2)	18 (100)	0.097^c^
	Left	5 (13.8)	0 (0)	
Location of stroke	Right	19 (52.8)	9 (50.0)	0.847^c^
	Left	17 (47.2)	9 (50.0)	
Brunnstrom upper extremity level (1 to 6)	Stage 1	1 (2.8)	0 (0)	0.846^c^
	Stage 2	3 (8.4)	2 (11.2)	
	Stage 3	2 (5.6)	1 (5.6)	
	Stage 4	9 (25.2)	4 (22.4)	
	Stage 5	7 (19.4)	6 (33.3)	
	Stage 6	14 (39.2)	5 (27.7)	
Brunnstrom lower extremity level (1 to 6)	Stage 3	2 (5.6)	1 (5.6)	0.658^c^
	Stage 4	11 (32.4)	5 (27.7)	
	Stage 5	5 (13.8)	4 (22.2)	
	Stage 6	18 (50.0)	8 (44.4)	

Abbreviations: APS, Anteroposteriore sway; cm, Centimeter; IQR, Interquartile range; kg, Kilogram; MLS, Mediolateral sway; mm, Millimeter; MMSE, Mini Mental State Examination; n, Number of people; SD, Standard deviation; X, Mean.

^a^ Student T Test.

^b^ Mann Whitney U Test.

^c^ Chi Square Test.

*p* < 0.05.

### 3.1 Inter‐ and Intrarater Reliability

The BBS was completed for all participants face‐to‐face and by teleassessment. The reliability results for the BBS obtained through synchronized and asynchronous teleassessments, focusing on interrater reliability, are presented in Table 2. Both the synchronized and asynchronous teleassessments demonstrated excellent interrater reliability. For the test group (*n* = 36), synchronized teleassessments demonstrated an ICC of 0.989 (95% CI: 0.978–0.994), with an SEM of 0.187, SEM95% of 0.367, and SDC95% of 0.517. The asynchronous teleassessments showed an ICC of 0.997 (95% CI: 0.994–0.998), SEM of 0.051, SEM95% of 0.100, and SDC95% of 0.141. For the retest group (*n* = 18), the synchronized teleassessments showed an ICC of 0.993 (95% CI: 0.982–0.997), SEM of 0.115, SEM95% of 0.225, and SDC95% of 0.318. The asynchronous teleassessments for this group showed an ICC of 0.988 (95% CI: 0.970–0.996), SEM of 0.190, SEM95% of 0.372, and SDC95% of 0.526 (Table 2).

**Table 2:** Inter‐rater reliability (ICC, SEM, SDC) for synchronous and asynchronous tele‐aassessments.

	Rater 1	Rater 2				
Test Group (n:36)	X ± SD	X ± SD	ICC (95% CI)	SEM	SEM95%	SDC95%
Synchronized tele‐assessment	47.2 ± 12.2	46.8 ± 11.8	0.989 (0.978–0.994)	0.187	0.367	0.517
Asynchronous tele‐assessment	47.3 ± 11.9	47.4 ± 11.9	0.997 (0.994–0.998)	0.051	0.100	0.141
Retest group (n:18)						
Synchronized tele‐assessment	46.5 ± 11.6	46.3 ± 11.7	0.993 (0.982–0.997)	0.115	0.225	0.318
Asynchronous tele‐assessment	47.4 ± 11.8	47.6 ± 11.3	0.988 (0.970–0.996	0.190	0.372	0.526

Abbreviations: CI, Confidence Interval; ICC, Intraclass Correlation Coefficient; SD, Standard Deviation; SDC, Smallest Detectable Change; SEM, Standart Error of Measurement; X, Mean.

**Figure 2:**
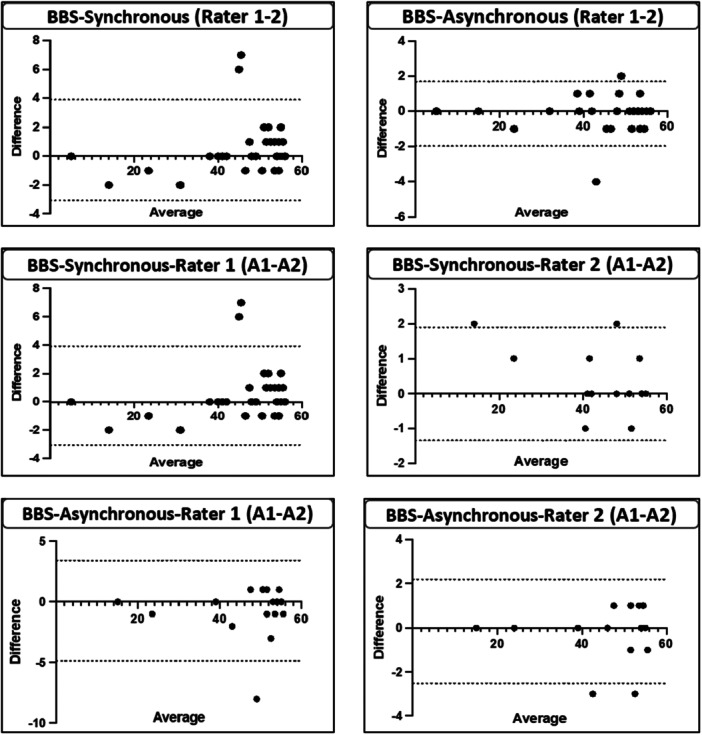
Bland–Altman plots illustrating intra and inter‐rater agreement and reliability. A1: 1st Assessment, A2: 2 nd Assessment. No systematic bias observed.

The intrarater reliability results of the BBS for the synchronized and asynchronous teleassessments are presented in Table 3. Both methods demonstrated excellent intrarater reliability across raters. For the synchronized teleassessments (*n* = 18), Rater 1 demonstrated an ICC of 0.986 (95% CI: 0.960–0.995) with an SEM of 0.216, SEM95% of 0.424, and SDC95% of 0.599. Similarly, Rater 2 demonstrated an ICC of 0.997 (95% CI: 0.993–0.999), SEM of 0.045, SEM95% of 0.088, and SDC95% of 0.124. For the asynchronous teleassessments (*n* = 18), Rater 1 demonstrated an ICC of 0.982 (95% CI: 0.952–0.993), SEM of 0.281, SEM95% of 0.551, and SDC95% of 0.779. Rater 2 demonstrated an ICC of 0.995 (95% CI: 0.986–0.995), SEM of 0.085, SEM95% of 0.167, and SDC95% of 0.236 (Table 3). Furthermore, a visual inspection of the Bland–Altman plots revealed no significant trend toward improving or worsening test performance (Figure 2).

**Table 3:** Intra‐rater reliabilty (ICC, SEM, SDC) for synchronous and asynchronous tele‐aassessments.

	Assessment 1	Assessment 2				
Synchronized tele‐assessment (n:18)	X ± SD	X ± SD	ICC (95% CI)	SEM	SEM95%	SDC95%
Rater 1	47.33 ± 11.74	46.50 ± 11.63	0.986 (0.960–0.995)	0.216	0.424	0.599
Rater 2	46.61 ± 11.30	46.33 ± 11.72	0.997 (0.993–0.999)	0.045	0.088	0.124
Asynchronous tele‐assessment (n:18)						
Rater 1	47.22 ± 11.31	47.94 ± 11.30	0.982 (0.952–0.993)	0.281	0.551	0.779
Rater 2	47.50 ± 11.33	47.66 ± 11.28	0.995 (0.986–0.995)	0.085	0.167	0.236

Abbreviations: CI, Confidence Interval; ICC, Intraclass Correlation Coefficient; SD, Standard Deviation; SDC, Smallest Detectable Change; SEM, Standard Error of Measurement; X, Mean.

### 3.2 Concurrent Validity

The concurrent validity results of the tests are shown in Table 4. There was a strong correlation between the BBS administered via teleassessment and the TBT and BBS administered face‐to‐face. There was a moderate correlation with postural sway data (Table 4).

**Table 4:** Correlation between tele‐assessment and face‐to‐face assessment.

	Berg balance scale‐synchronized			Berg balance scale‐asynchronous		
Variables (n:36)	*r*	95% CI	*p*	*r*	95% CI	*p*
Berg Balance Scale‐Face to Face	0.970	0.944–0.984	< 0.001	0.945	0.884–0.975	< 0.001
Tinetti Balance Test‐Face to Face	0.901	0.835–0.948	< 0.001	0.885	0.792–0.947	< 0.001
Mediolateral sway (mm)	−0.406	−0.651 to −0.128	0.014	−0.402	−0.655 to −0.089	0.027
Anteroposterior sway (mm)	−0.457	−0.696 to −0.168	0.005	−0.487	−0.728 to −0.173	0.003
Sway velocity (mm/s)	−0.541	−0.782 to −0.261	0.001	−0.538	−0.780 to −0.225	0.001

Abbreviations: r, Spearman Correlation Coefficient; CI, Confidence interval; mm, Millimeter; s, Second. *p* < 0.05.

## 4 Discussion

Our findings suggest promising intra‐ and interrater reliability for both teleassessment methods, indicating their potential utility for remote assessments of balance. Additionally, a notable correlation was identified between BBS scores obtained through teleassessment and traditional face‐to‐face methods, supporting the preliminary validity of these approaches. Although these results indicate the feasibility of applying the BBS through teleassessment as an alternative to face‐to‐face assessments, they should be interpreted with caution due to the study's limitations. Further research involving larger populations is needed to validate the findings and enhance their generalizability.

Compared to national averages in Türkiye (approximately 65–70 years), the relatively younger age of our study (mean age 55 years) can be attributed to the study's specific inclusion criteria and design features [39]. Participation required individuals to engage in tele‐assessment procedures, including performing video recordings, using internet‐based platforms, and safely conducting functional balance tests at home. These prerequisites likely favored the recruitment of younger, more independent stroke survivors with higher functional capacity and fewer comorbidities. Consistent with our findings, previous telehealth and telerehabilitation studies have also reported a tendency to recruit younger participants, attributed to the technological literacy requirements and the physical demands associated with remote assessment protocols.

Durfee et al., in their study in which they explored the technical feasibility of teleassessments for rehabilitation in healthy individuals, examined the suitability of the BBS for teleassessment [40]. Additionally, Venkataraman et al., in their study with veterans, examined the suitability of the BBS for teleassessment using videos with different transmission characteristics. They reported that although some information was lost when using videos, it was still possible to reliably implement the BBS remotely in standard clinical settings [26]. Gillespie et al. investigated the reliability of synchronous teleassessments of the BBS in stroke patients. These teleassessments, conducted with a therapy assistant present in the patients’ homes to assist with setup and implementation, demonstrated high reliability and close alignment with in‐person evaluations in terms of accuracy and feasibility. In contrast to Gillespie et al., our study included asynchronous assessments but relied entirely on family members and caregivers for home setup and implementation, without the involvement of a therapy assistant [27].

In studies in which the validity and reliability of the BBS applied face‐to‐face to stroke patients were investigated, it was generally found that the scale was good to excellent. Berg et al. as a result of their evaluation of 70 acute stroke patients at 2, 4, 6, and 12 weeks, found intrarater and interrater reliability to be excellent (ICC 0.98 and 0.97) [41]. In Mao et al.'s study, 123 stroke patients were evaluated on days 30, 90, and 180 and the ICC score found was 0.95 [42]. The ICC results we obtained in our study are similar to those in the literature. In our study, SEM and SDC95% values were calculated to determine the reliability and clinical applicability of the measurement tools. SEM establishes reliability by reflecting the amount of error in a measurement instrument and defines the range around the measured value within which the theoretical “true” value can be found [43]. SDC95% refers to the minimum amount of change required to guarantee that the observed change is real and not due to measurement error. Therefore, SDC95% can be used as an important reference for classifying individuals as “changed” or “unchanged” [44]. In our study, the low values of SEM and SDC95% indicate that both teleassessment methods have low measurement errors and provide high sensitivity in detecting small changes. According to the measurement results in our study, the true value of a BBS score obtained by teleassessment lies within a narrow range around the measured value with 95% probability. This suggests that teleassessment may be suitable for clinical use, especially for monitoring and evaluating minimal progress during rehabilitation. The lower SDC values observed in our study compared to previous literature likely result from the high reliability (ICC: 0.988–0.997) and low measurement variability within our sample. The use of teleassessment methods, particularly asynchronous video recordings, may have reduced measurement errors by standardizing assessment conditions. Additionally, the inclusion of younger, higher‐functioning stroke patients likely contributed to this reduced variability, leading to smaller SDC estimates. Future studies involving more diverse stroke populations and varied assessment environments are recommended to confirm and generalize these findings.

Chiaramonte et al. found a high level of correlation (*r* = 0.85) between the TBT and the BBS [45]. Their study differs from ours in that both balance assessments were applied in the clinic. However, when the study results are examined, it is seen that they obtained results similar to those in our study. We determined a high level of correlation between the TBT applied face‐to‐face and the BBS applied via teleassessment. In a study reported in the literature, a moderate significant negative correlation was found between the BBS total score and center of gravity swing velocity, weaker but significant negative correlations were detected between the BBS total score and MLS, and, finally, a moderate negative correlation was observed between the BBS total score and APS [46]. Corriveau et al. in their study involving stroke patients, the relationship between APS and MLS and balance scales was examined. They found that BBS score had a moderate correlation with APS (*r* = −0.57) and a moderate correlation with MLS (*r* = −0.53) [47]. We similarly found a moderate negative correlation between postural sway data and BBS score. An important result of our study was that the BBS applied both synchronously and asynchronously was compatible with the balance platform. Our finding of a moderate correlation with the balance platform, which is frequently used to evaluate balance in stroke patients, suggests that both teleassessment methods are successful in assessing balance. The validity of the asynchronous assessment is an important result of our study. Although the asynchronous assessment requires the Internet to send videos, a continuous Internet connection is not necessary, which is an important advantage of this method. Especially in regions with poor Internet infrastructure, the asynchronous teleassessment approach may enable telerehabilitation services to reach a wider audience. Therefore, asynchronous teleassessment offers a feasible and effective solution for patients with limited Internet connectivity.

In addition, we examined the reliability and validity of the TBT previously. The results we obtained for the TBT in that study were similar to those in the present study [22]. In the current study, we found a moderate to very strong relationship between the BBS administered via teleassessment and balance assessments administered face‐to‐face. Our findings were generally similar to those in our previous study examining the suitability of the TBT for teleassessment. While the BBS when applied only by teleassessment was moderately related to mediolateral balance assessments, the TBT applied via teleassessment was insignificantly related. For this reason, we think that it may be more advantageous to use the BBS in individuals with mediolateral balance problems.

### 4.1 Limitations

Since the stroke patients were evaluated more than once with 3 different methods in our study, it is likely that a learning effect developed. Therefore, to minimize the learning effect, the order in which different evaluation methods were applied to each new patient was changed and attempts were made to reduce negative effect of this limitation. Our study sample consisted of individuals with relatively high education levels living in urban areas. Including individuals with low education and income levels living in rural areas could have enabled us to obtain more inclusive results, which is another important limitation of our study. Another limitation is that the study sample was quite young (mean 55.5 years) and the time since the acute event was quite long. A significant limitation of our study is the relatively small sample size of 18 participants for evaluating intrarater reliability. Collectively, these limitations affect the generalizability of the study results and highlight the need for future research with larger and more diverse populations.

### 4.2 Recommendations

During the study, we encountered some challenges in a few assessments, particularly related to asynchronous video recordings. In several cases, issues such as suboptimal video quality, incomplete recordings, or camera misalignment were observed, which may have influenced the ease of evaluation. These situations were more common among participants who did not have younger or more technologically experienced caregivers to assist with the recording process. Similarly, during synchronous assessments, minor internet disruptions occurred in two cases, briefly affecting session flow. While these challenges did not significantly impact the overall feasibility of the study, they highlight important considerations for future research. Providing clear recording instructions, offering caregiver support, and ensuring technical preparedness may help enhance the quality and reliability of teleassessment procedures, especially in populations with varying levels of access to digital resources.

## 5 Conclusion

The findings of the present study suggest that the BBS demonstrates promising reliability and validity for assessing balance in chronic stroke patients through teleassessment. The results provide preliminary evidence supporting the potential feasibility of both synchronous and asynchronous teleassessment for balance evaluation in this population. However, these findings should be interpreted with caution due to the study's limitations, and further research with larger and more diverse samples is required to confirm these results and improve their generalizability.

## Author Contributions

Idea/Concept: Birol Önal, Nezire Köse; Design: Birol Önal, Nezire Köse; Control/Supervision: Nezire Köse; Hatice Yağmur Zengin; Data Collection and/or Processing: Hatice Yağmur Zengin; Analysis and/or Interpretation: Birol Önal, Hatice Yağmur Zengin; Literature Review: Birol Önal, Şeyma Nur Önal; Writing the Article: Birol Önal, Nezire Köse, Şeyma Nur Önal; References: Birol Önal, Şeyma Nur Önal.

## Ethics Statement

The study was approved by the local ethical committees in all participating centers and was carried out according to the declaration of Helsinki.

## Conflicts of Interest

The authors declare no conflicts of interest.

## Data Availability

The data that support the findings of this study are available from the corresponding author, [B.Ö.], upon reasonable request.
